# Aberrant resting-state functional connectivity and topological properties of the subcortical network in functional dyspepsia patients

**DOI:** 10.3389/fnmol.2022.1001557

**Published:** 2022-10-13

**Authors:** Pan Zhang, Zhaoxuan He, Yangke Mao, Ruirui Sun, Yuzhu Qu, Li Chen, Peihong Ma, Shuai Yin, Tao Yin, Fang Zeng

**Affiliations:** ^1^Acupuncture and Tuina School, Chengdu University of Traditional Chinese Medicine, Chengdu, China; ^2^Acupuncture and Brain Science Research Center, Chengdu University of Traditional Chinese Medicine, Chengdu, China; ^3^School of Acupuncture-Moxibustion and Tuina, Beijing University of Chinese Medicine, Beijing, China; ^4^First Affiliated Hospital, Henan University of Traditional Chinese Medicine, Zhengzhou, Henan, China

**Keywords:** functional dyspepsia, subcortical network, resting-state functional connectivity, graph theory, functional magnetic resonance imaging

## Abstract

Functional dyspepsia (FD) is a disorder of gut-brain interaction. Previous studies have demonstrated a wide range of abnormalities in functional brain activity and connectivity patterns in FD. However, the connectivity pattern of the subcortical network (SCN), which is a hub of visceral information transmission and processing, remains unclear in FD patients. The study compared the resting-state functional connectivity (rsFC) and the global and nodal topological properties of SCN between 109 FD patients and 98 healthy controls, and then explored the correlations between the connectivity metrics and clinical symptoms in FD patients. The results demonstrated that FD patients manifested the increased rsFC in seventeen edges among the SCN, decreased small-worldness and local efficiency in SCN, as well as increased nodal efficiency and nodal degree centrality in the anterior thalamus than healthy controls (*p* < 0.05, false discovery rate corrected). Moreover, the rsFC of the right anterior thalamus-left nucleus accumbens edge was significantly correlated with the NDSI scores (*r* = 0.255, *p* = 0.008, uncorrected) and NDLQI scores (*r* = −0.241, *p* = 0.013, uncorrected), the nodal efficiency of right anterior thalamus was significantly correlated with NDLQI scores (*r* = 0.204, *p* = 0.036, uncorrected) in FD patients. This study indicated the abnormal rsFC pattern, as well as global and nodal topological properties of the SCN, especially the bilateral anterior thalamus in FD patients, which enhanced our understanding of the central pathophysiology of FD and will lay the foundation for the objective diagnosis of FD and the development of new therapies.

## Introduction

Functional dyspepsia (FD) is one of the most common functional gastrointestinal disorders (FGIDs). According to the consensus of the Rome committee, FD refers to a group of upper gastrointestinal syndromes (e.g., early satiation, post-prandial fullness, epigastric pain, epigastric burning, etc.), which could not be explained by organic, systemic, or metabolic reasons (Carbone et al., 2020). It was reported that the global prevalence of FD ranged from 5 to 30% (Ford et al., 2020). Although FD is not a life-threatened disease, it significantly affects the quality of life (QoL) of the patients and causes high medical costs (Miwa et al., 2022). Therefore, clarifying the pathophysiology of FD, making an accurate diagnosis, and developing effective therapies for FD have become major concerns for FD researchers.

Functional dyspepsia is officially defined as a disorder of brain-gut interaction in the latest Rome diagnostic criteria (Drossman et al., 2018), which provides a new perspective to reveal the pathophysiology of FD from the central nervous system. Evidence from neuroimaging studies indicated that FD patients manifested significant alterations in multiple brain regions, including the thalamus, amygdala, hippocampus, etc. (Liu et al., 2014; Lee et al., 2016; Kano et al., 2018; Skrobisz et al., 2019), which were the essential components of the subcortical networks (SCN). For instance, Van Oudenhove et al. (2010) reported the correlation between gastric hypersensitivity and FD patients with abuse history, and they found abuse history was associated with brain activity differences in the hippocampus and amygdala; Liu et al. (2014) found that the gray matter volumes of the thalamus, putamen, and caudate in FD patients significantly differed from those of healthy controls (HC); Nan et al. (2015) demonstrated that FD patients manifested increased fractional anisotropy in the thalamic than HC. In another study, Xu et al. (2021) found the white matter functional connectome of FD patients showed decreased nodal efficiency in the left posterior thalamic radiation than HC. Moreover, our recent independent components analysis-based study indicated that the thalamus-related resting state functional connectivity (rsFC) could be the critical feature to discriminate FD patients from HC (Yin et al., 2021b). All these findings together suggested that the alteration of the SCN might play an important role in the central pathophysiology of FD. However, due to the lack of a finely segmented brain atlas of subcortical regions, the connectivity patterns and topological features of the SCN in FD patients were still unclear, which limited the further understanding of the central pathophysiological alterations among FD patients.

Recently, a detailed atlas for the segmentation of the subcortex regions was proposed (Tian et al., 2020), which provided an important step toward a better understanding of the connectivity patterns of SCN. Based on this subcortex atlas, the current study aimed to investigate both the rsFC pattern and topological properties of the SCN and further explored the correlations between the aberrant connectomics features and clinical symptoms in FD patients. The results of this study will promote the understanding of the central pathophysiology of FD, and lay the foundation for the objective diagnosis and development of novel therapies for FD from the perspective of brain connectivity.

## Materials and methods

### Participants

One hundred and twenty FD patients and 103 HC were enrolled. The FD patients were recruited from the Hospital of Chengdu University of Traditional Chinese Medicine (CDUTCM) and the campus of CDUTCM from March 2016 to May 2018. After being diagnosed by 2 experienced gastroenterologists according to Rome IV Criteria for FD (Drossman, 2016) and undergoing physical and laboratory examinations, the potential FD patients were enrolled if they met all the following inclusion criteria: (a) were right-handed and aged from 18 to 40 years; (b) did not take any gastrointestinal prokinetic agent in the 2 weeks before enrollment; (c) did not take part in any other clinical trials; and (d) signed informed consent. Patients were excluded if they (a) were pregnant or lactating, or intended to be pregnant in the 6 months, or (b) were complicated with other FGIDs such as irritable bowel syndrome or functional constipation, or (c) had a history of psychiatric and neurological disorders or head trauma with loss of consciousness, or (d) accompanied with other organic diseases, or (e) had any contraindication to functional magnetic resonance imaging (fMRI) scannings, such as a cardiac pacemaker, defibrillator, metal stent, or electronic implant.

The right-handed HC (aged from 18 to 40 years) without a history of gastrointestinal, neurological, or psychiatric disorders were recruited from the campus of CDUTCM. They underwent a basic evaluation including a review of medical history, physical examinations, and laboratory tests to exclude potential diseases.

### Clinical evaluation

The Nepean Dyspepsia Symptom Index (NDSI) and Nepean Dyspepsia Life Quality Index (NDLQI) were used to assess the severity of clinical symptoms and the QoL of FD patients, respectively (Talley et al., 1999).

### Functional magnetic resonance imaging data acquisition

The resting-state MRI data were acquired *via* a 3.0 T magnetic resonance scanner (Siemens, Munich, Germany) with an eight-channel-phased array head coil at Huaxi Magnetic Resonance Research Center of the West China Hospital. Parameters of the high-resolution 3-dimensional T1-weighted imaging were as follows: repetition time = 1900 ms, echo time = 2.26 ms, flip angle = 7°, slice thickness = 1 mm, slice number = 156, matrix size = 128 × 128, and field of view = 256 × 256 mm^2^. The blood oxygen level-dependent fMRI data were acquired with the echo-planar imaging: repetition time = 2000 ms, echo time = 30 ms, flip angle = 90°, slice number = 30, slice order = interleaved, matrix size = 64 × 64, field of view = 240 × 240 mm^2^, slice thickness = 5 mm, time points = 180. During the entire scanning procedure, patients were asked to keep their eyes closed and their ears plugged.

### Image preprocessing and head motion control

The fMRI data preprocessing was performed with SPM12^1^ and DPARSF 4.1 toolbox^2^ (Yan et al., 2016) based on MATLAB 2017b. First, the first 10 time points were removed, allowing for signal stabilization. Then, the remaining images were corrected for slice-timing and head motion, and the corrected images were normalized to the Montreal Neurologic Institute space and smoothed with a 4 mm Gaussian kernel of full width at half maximum.

As excessive head motion significantly impacts the reliability of brain network analysis (Mahadevan et al., 2021), strict head motion control criteria were applied in this study (Jenkinson et al., 2002; Yan et al., 2013). Accordingly, participants were excluded if the mean framewise displacement was over 0.2 mm and the max head motion over 1.5 mm or 1.5 degrees. In addition, following the correction strategy recommend by recent studies (Raitamaa et al., 2021; Wang et al., 2021), the six rigid-body motion parameters and signals of white matter and CSF were regressed using the component-based noise correction method to minimize the influences of head motion on the estimation of the rsFC matrices.

### Network construction

According to the subcortex atlas (Tian et al., 2020), the subcortex was divided into 16 components (Supplementary material 1), and each component represented a node in the SCN. The mean time series of nodes were obtained by averaging the fMRI time series of each component, and Pearson’s correlation coefficients were then calculated between the mean time series of each pair of nodes to generate the 16 × 16 correlation matrix. Subsequently, Fisher’s *r*-to-*z* transformation was applied to convert correlation coefficients to *Z*-values for improving normality, thus the SCN matrix was constructed for each participant.

### Graph theory analysis

To characterize the topological properties of the SCN, graph-theoretic analysis was conducted with the Graph Theoretical Network Analysis toolbox (GRETNA) 2.0 toolbox^3^ (Wang et al., 2015). First, the constructed SCN rsFC matrix was post-processed with the following three steps: (1) sparsity all matrices with the thresholds of 0.1 to 0.35 (step = 0.05) (Tu et al., 2019; Yin et al., 2021a), and thresholded each symmetrical matrix into a binarized matrix with a fixed sparsity value, and only positive connections were analyzed, and then six binary matrixes were created; (2) generate benchmark random networks that match real SCN with respect to the number of nodes and edges and degree distribution; and (3) calculate graph-based global and nodal network metrics, and the details and interpretations of global and nodal topological properties were described in Supplementary material 2. Second, the integrated area under the curve (AUC) value of all related network metrics over the corresponding sparsity range (0.1 ≤ range ≤ 0.35, step = 0.05) was calculated to explore the SCN topological properties differences between FD patients and HC. Third, the results of the graph-theoretic analysis were displayed *via* BrainNet Viewer software^4^ (Xia et al., 2013).

### Statistical analysis

SPSS 25.0 (SPSS Inc., Chicago, IL, USA) software was used for the demographic analysis between FD and HC groups. Student’s *t*-tests, Mann–Whitney *U* test, and χ^2^ test were applied to the analysis of continuous and normally distributed data, non-normally distributed data, and dichotomous variable (the gender), respectively.

With age, gender, and mean framewise displacement as covariates, analysis of covariance (ANCOVA) was applied to compare the between-group differences in rsFCs and topological properties. Moreover, to investigate the correlations between the clinical measures and neuroimaging metrics that showed group differences in FD patients, the partial correlation analyses were performed with age, gender, and mean framewise displacement as covariates. The significance thresholds for the between-group comparisons and correlation analyses were set to 2-tailed *p* < 0.05, and the false discovery rate (FDR) was applied for the multiple comparison correction.

## Results

### Demographic and clinical characteristics

Eleven FD patients and 5 HC were excluded for the excessive head motion. Therefore, 109 FD patients and 98 HC were finally included in the data analysis. There were no significant differences between FD patients and HC in demographic characteristics (age, gender, height, weight, Table 1).

**TABLE 1:** The demographic and clinical characteristics of the eligible FD patients and HC.

	FD patients	HC	Statistic	*p*-value
Gender (F/M)	90/19	78/20	*χ^2^* = 0.299	*p* = 0.584
Age (Y)	22.16 ± 2.16	20.85 ± 2.06	*t* = 4.451	*p* < 0.001*
Height (cm)	161.19 ± 7.39	162.41 ± 6.58	*t* = −1.244	*p* = 0.215
Weight (kg)	51.90 ± 8.27	52.70 ± 7.48	*t* = −0.733	*p* = 0.464
Duration (months)	39.17 ± 31.29	N/A	N/A	N/A
NDSI	42.57 ± 15.30	N/A	N/A	N/A
NDLQI	76.97 ± 10.12	N/A	N/A	N/A

FD, functional dyspepsia; HC, healthy controls; F/M, female/male; Y, year; SID, Symptom Index of Dyspepsia; NDLQI, Nepean Dyspepsia Life Quality Index; SAS, Self-Rating Anxiety Scale; SDS, Self-Rating Depression Scale; N/A, not applicable.

*The marker indicated a significant difference between the FD group and the HC group.

### Between-group differences in the resting-state functional connectivity

**FIGURE 1:**
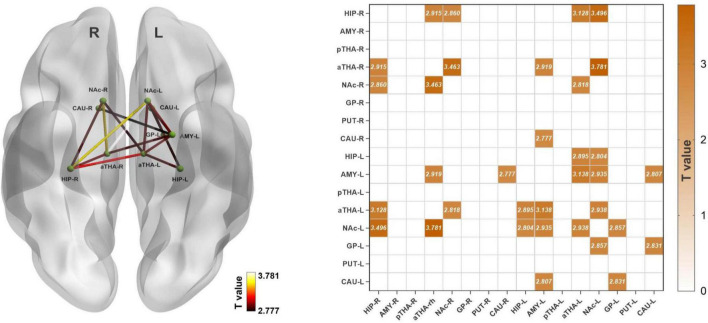
Alterations of the rsFC in subcortical networks in FD patients. FD patients showed seventeen increased rsFC edges (*p*_FDR_ < 0.05). Abbreviations: rsFC, resting-state functional connectivity; R, right; L, left; HIP_R, right hippocampus; AMY_R, right amygdala; pTHA_R, right posterior thalamus; aTHA_R, right anterior thalamus; NAc_R, right nucleus accumbens; GP_R, right globus pallidus; PUT_R, right putamen; CAU_R, right caudate nucleus; HIP_L, left hippocampus; AMY_L, left amygdala; pTHA_L, left posterior thalamus; aTHA_L, left anterior thalamus; NAc_L, left nucleus accumbens; GP_L, left globus pallidus; PUT_L, left putamen; CAU_L, left caudate nucleus. The coordinate of the subcortical regions was provided in Supplementary material 1.

Compared to the HC, FD patients manifested stronger rsFC in seventeen edges among the SCN, which many involved in the bilateral anterior thalamus (aTHA), right hippocampus (HIP_R), and left nucleus accumbens (NAc_L), bilateral amygdala (AMY), and left caudate nucleus (CAU_L), etc. (*p*_*FDR*_ < 0.05, 120 times tests) (Figure 1 and Table 2).

**TABLE 2:** Between-group differences in the rsFC.

rsFC	Statistic	*p*-value
aTHA_R-HIP_R	*t* = 2.915	*p* = 0.004*
NAc_R-HIP_R	*t* = 2.860	*p* = 0.005*
aTHA_L-HIP_R	*t* = 3.128	*p* = 0.002*
NAc_L-HIP_R	*t* = 3.946	*p* = 0.001*
HIP_R-aTHA_R	*t* = 2.915	*p* = 0.001*
NAc_R-aTHA_R	*t* = 3.463	*p* = 0.004*
AMY_L-aTHA_L	*t* = 2.919	*p* = 0.000*
NAc_L-aTHA_R	*t* = 3.781	*p* = 0.005*
AMY_L-CAU_R	*t* = 2.777	*p* = 0.006*
aTHA_L-HIP_L	*t* = 2.895	*p* = 0.004*
NAc_L-HIP_L	*t* = 2.804	*p* = 0.006*
aTHA_L-AMY_L	*t* = 3.138	*p* = 0.002*
NAc_L-AMY_L	*t* = 2.935	*p* = 0.004*
CAU_L-AMY_R	*t* = 2.807	*p* = 0.005*
NAc_L-aTHA_L	*t* = 2.938	*p* = 0.004*
NAc_L-GP_L	*t* = 2.857	*p* = 0.005*
CAU_L-GP_R	*t* = 2.831	*p* = 0.005*

rsFC, rest state functional connectivity; HIP_R, right hippocampus; AMY_R, right amygdala; pTHA_R, right posterior thalamus; aTHA_R, right anterior thalamus; NAc-R, right nucleus accumbens; GP-R, right globus pallidus; PUT-R, right putamen; CAU_R, right caudate nucleus; HIP_L, left hippocampus; AMY_L, left amygdala; pTHA_L, left posterior thalamus; aTHA_L, left anterior thalamus; NAc_L, left nucleus accumbens; GP_L, left globus pallidus; PUT_L, left putamen; CAU_L, left caudate nucleus.

*The marker indicated a significant difference between the FD group and the HC group.

### Between-group differences in the global topological properties

**FIGURE 2:**
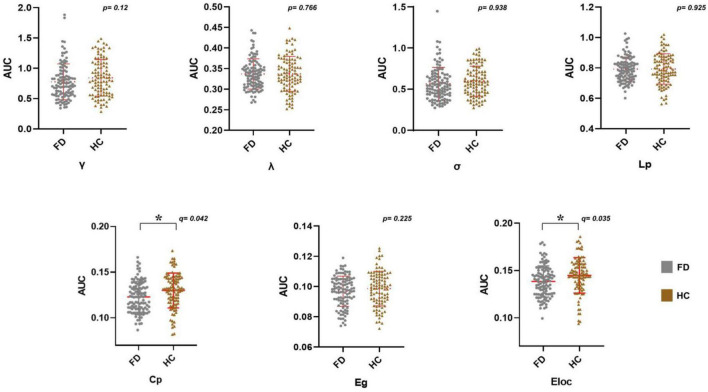
Alterations in the AUC of global network metrics in FD patients. FD patients showed a significantly lower *Cp* and *Eloc* (*p_FDR_ < 0.05). Abbreviations: AUC, area under the curve; FD, functional dyspepsia; HC, health controls; γ, normalized clustering coefficient; λ, normalized characteristic path length; σ, small worldness; Cp, clustering coefficient; Lp, characteristic path length; Eglob, global efficiency; Eloc, local efficiency; FDR, false discovery rate.

Both FD patients and HC showed typical features of the small-world properties in the SCN [normalized clustering coefficient (γ) > 1, normalized shortest path length (λ) ≈ 1, and small-worldness (σ) > 1] (Supplementary material 4). Among the seven global topological properties, the clustering coefficient (*Cp*) and local efficiency (*Eloc*) of FD were significantly lower than HC (*p_*FDR*_* < 0.05, 7 times tests) (Figure 2).

### Between-group differences in the regional topological properties

**FIGURE 3:**
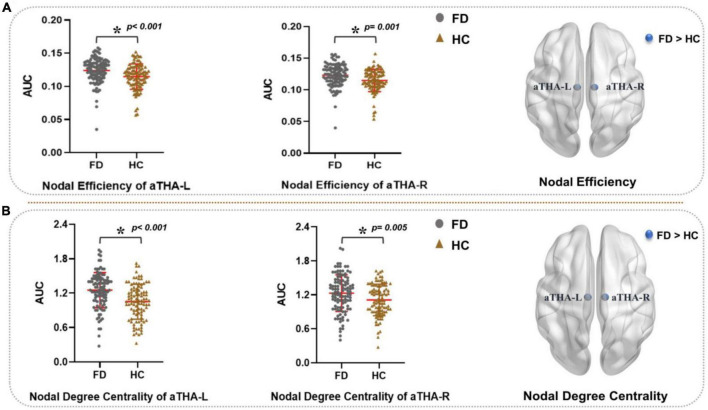
Alterations in the AUC of nodal properties in FD patients (*p*_FDR_ < 0.05). FD patients showed: **(A)** higher nodal efficiency and **(B)** nodal degree centrality in aTHA. Abbreviations: L, left; R, right; AUC, area under the curve; FDR, false discovery rate; FD, functional dyspepsia; HC, healthy controls; aTHA-rh, anterior right head of thalamus; aTHA-lh, anterior left head of thalamus.

Compared with HC, FD patients showed a significantly higher nodal efficiency (Figure 3A and Table 2) and nodal degree centrality (Figure 3B and Table 3) in the aTHA-R and the aTHA-L (*p_*FDR*_* < 0.05, 2 times tests).

**TABLE 3:** Between-group differences in the regional topological properties.

Parameters	Brain regions	FD Mean ± SD	HC Mean ± SD	Statistic	*p*-value
**Nodal efficiency**					
FD > HC	aTHA_R	0.123 ± 0.018	0.115 ± 0.017	*t* = 3.34	*p* = 0.001*
	aTHA_L	0.124 ± 0.019	0.114 ± 0.018	*t* = 3.596	*p* < 0.001*
**Nodal degree centrality**					
FD > HC	aTHA_R	1.228 ± 0.324	1.108 ± 0.279	*t* = 2.838	*p* = 0.005*
	aTHA_L	1.251 ± 0.307	1.051 ± 0.306	*t* = 4.681	*p* < 0.001*

FD, functional dyspepsia; HC, healthy controls; aTHA_R, right anterior thalamus; aTHA_L, left anterior thalamus.

*The marker indicated a significant difference between the FD group and the HC group. *p* < 0.05, FDR corrected.

### Correlations of connectivity metrics and clinical symptoms

**FIGURE 4:**
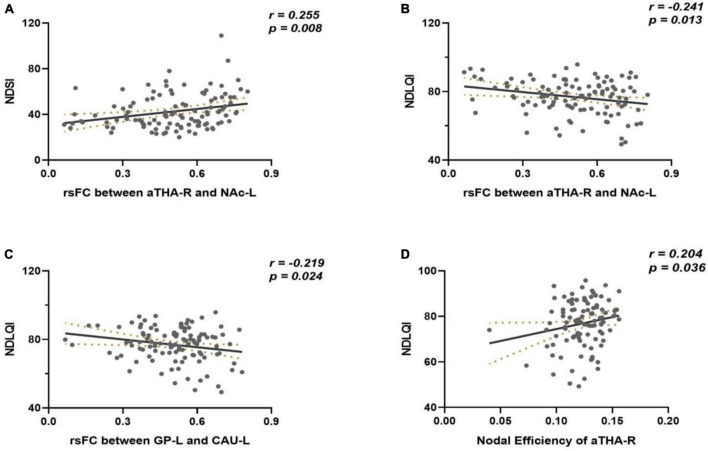
Results of correlation analyses. **(A)** the rsFC of the aTHA_RNAC_L edge was positively correlated with the NDSI; **(B,C)** the rsFC of the aTHA_R-NAc_L edge and GP_L-CAU_L edge were negatively correlated with the NDLQI; **(D)** the nodal efficiency of aTHA_R was positively correlated with the NDLQI.

There was no significant correlation between the connectivity metrics and clinical symptoms in FD patients under the threshold of *p*_*FDR*_ < 0.05 (42 times tests). However, under the uncorrected threshold, the rsFC of the aTHA_R-NAC_L edge was positively correlated with the NDSI (*r* = 0.255, *p* = 0.008) (Figure 4A), the rsFC of the aTHA_R-NAc_L edge (*r* = −0.241, *p* = 0.013) and GP_L-CAU_L edge (*r* = −0.219, *p* = 0.024) were negatively correlated with the NDLQI (Figures 4B,C), and the nodal efficiency of aTHA_R was positively correlated with the NDLQI (*r* = 0.204, *p* = 0.036) (Figure 4D).

## Discussion

With a relatively large sample size, this study explored the rsFC pattern and the topological organization of SCN in FD patients for the first time. The results revealed that FD patients had abnormally increased rsFC, as well as aberrant global and nodal topological characteristics within the SCN, especially the bilateral aTHA.

The subcortex region mainly included the thalamus, basal ganglia nucleus, hippocampus, etc. For its special location (between the brainstem/medulla and the cerebral cortex), the subcortical region mainly serves as a relay station for the transmission of peripheral signals to the cortical regions. In general, afferent visceral signals conveyed from the gastroduodenal projected to brain areas related to visceral sensorimotor processing such as the thalamus, basal ganglia, somatosensory cortex, etc. (Hunt and Mantyh, 2001; Critchley and Harrison, 2013; Moloney et al., 2015). Repeated attacks of epigastric pain or burning, post-prandial fullness, or early satiety are the typical clinical symptoms of FD, all of which are associated with abnormal visceral sensations (Black et al., 2020). Due to the recurrent attacks of these hypersensitive visceral stimuli from the gastroduodenal, the nuclei of SCN repeatedly receive these interoceptive signals and then project these signals to the higher visceral sensorimotor center, which may cause hyperactivation of the subcortical nuclei and change in integration between connected nuclei within the SCN (Drewes et al., 2020). The Hyperactivation of subcortical nuclei was found in FD patients, as well as the strengthened rsFCs of subcortical areas such as the thalamus-Para hippocampus/hippocampus (Lee et al., 2016; Yin et al., 2021b). In the present study, the strengthened rsFC of the aTHA_R-NAc_L edge and GP_L-CAU_L edge showed a tendency to correlate with the symptom severity or QoL. Since gastrointestinal hypersensitivity is thought to be important in generating symptoms in FD, which may be caused by central sensitization (Suciu et al., 2019), and some subcortical nuclei (aTHA, NAc, GP, CAU, etc.) have been reported to be involved in the processing of aberrant sensations from the gastrointestinal tract (Lu et al., 2004; Wang et al., 2009). Thus, our findings may suggested that the strengthed rsFCs between interconnected connected subcortical nuclei were the manifestations of central sensitization, contributing to the occurrence of FD. Similarly, the strengthened rsFCs of subcortical were also found in the other FGIDs like irritable bowel syndrome, which manifested gastrointestinal hypersensitivity either (Osadchiy et al., 2020). These findings corroborated with previous studies that the subcortex was involved in the pathophysiology of FD and enriched the evidence of central sensitization of abnormal affective responses to visceral afferent sensory information in FD. It should be emphasized that the correlation analysis’s findings were not statistically significant after the FDR correction. Therefore, more research is required to determine whether these rsFCs contribute to the pathophysiology of FD.

Functional dyspepsia patients also manifested aberrant global topological properties of SCN. Compared with HC, FD patients showed a significantly decreased *Cp* of the SCN but no significant change in the shortest path length (*Lp*). Within a network, the *Cp* reflects the cliquishness or local specialization of a typical neighborhood and the *Lp* characterizes global integration (Li et al., 2014; Zhao et al., 2019), decreased *Cp* means poor local connectivity of nodes within a network. Thus, our findings supported disrupted SCN function of FD patients and revealed FD patients had a decreased ability for specialized processing while maintaining the effectiveness of global integration. Moreover, FD patients also showed decreased *Eloc*, which reflected a higher cost of information transfer within SCN (Yun and Kim, 2021). In addition, both *Cp* and *Eloc* can reflect the ability for specialized processing to occur within densely interconnected groups of brain regions (Seguin et al., 2018), and the decreased *Cp* and *Eloc* among FD patients would lead to the loss of optimal balance of the local network information processing and efficient overall routing with shortcuts of SCN (Latora and Marchiori, 2001; Liu et al., 2008). Further, apart from the aberrant topological properties in the SCN, FD patients also presented whole-brain network topology alterations (Nan et al., 2016). Interestingly, the altered topological properties of the brain network were also found in other FGIDs such as irritable bowel syndrome and functional constipation (Qi et al., 2016; Liu et al., 2021). Together, these findings supported that altered topological properties of brain networks are important central pathophysiological features of FGIDs, which may imply adaptive mechanisms for maintaining balance in the human body.

Additionally, apart from the altered bilateral aTHA rsFC coupling pattern in SCN, we also found the altered nodal topology properties of the bilateral aTHA. As a core node of SCN, the thalamus integrates and relays the nociceptive and homeostatic information to higher centers of the brain (Elvsåshagen et al., 2021). Moreover, it also interacts with descending pathways that modulate visceral sensations and is crucial for the bidirectional communication between the gastrointestinal tract and the brain (Weltens et al., 2018; Roy et al., 2022). In the new parcelation hierarchy of the subcortex, the thalamus was divided into the aTHA and the posterior thalamus (Tian et al., 2020), and the aTHA was considered a trigger for memory, cognition, and perception processing (Nelson, 2021; Sweeney-Reed et al., 2021). In this study, FD patients showed higher nodal efficiency in the aTHA and the nodal efficiency of aTHA-R showed a positive correlation tendency with NDLQI, while the nodal efficiency measures the ability of information propagation between a node and the remaining nodes in the network (Rubinov and Sporns, 2010; Ma et al., 2018). A plausible explanation for these findings is nociceptive signals repeatedly from the gastrointestinal tract have an impact on the central processing visceral sensation ability of the aTHA, which may result in the somatic memories reinforcement of aTHA to the nociceptive afferent stimuli from the gastrointestinal tract and increase compensational information propagation ability of aTHA to maintaining the communication between gut and brain, that is causing the changes of aTHA nodal efficiency. However, the correlation between aTHA and symptoms needs to be further verified, it should be highlighted, since there was no statistically significant result after multiple comparison corrections.

Some limitations should be mentioned in this study. Firstly, using 7T fMRI can yield a more detailed atlas of the subcortex, but we only have 3T MRI data, which impedes further exploration of the central pathological of FD based on a finer subcortex parcelation. Second, all participants were Chinese, so applicability to other ethnic/racial and cultural contexts must be confirmed. Third, there are gender differences in FD prevalence (Zand Irani et al., 2021), thus more females with FD were included in this study, although we have regressed gender as a covariate, this still could not completely circumvent the effect of gender bias on the results.

## Conclusion

In conclusion, the current study compared the rsFC pattern and topologic properties of SCN between FD patients and HC. The results detected that FD patients manifested increased rsFC in seventeen edges among the SCN, as well as increased nodal topological properties in the bilateral aTHA but decreased global topological properties in the SCN. These findings expanded our understanding of the central pathological characteristics of FD from the perspective of altered SCN. Additionally, some advanced methods, like machine learning or manifold learning, have been successfully applied to find biomarkers for some diseases, such as physical pain and schizophrenia (Wager et al., 2013; Gallos et al., 2021a,b). In the near future, these methods could also be used to identify biomarkers for the diagnosis of FD at the node or rsFC level, which will lay the ground work for the identification of FD and promote the development of novel neuromodulation methods for treating FD.

## Data availability statement

The original contributions presented in this study are included in the article/Supplementary material, further inquiries can be directed to the corresponding authors.

## Ethics statement

The studies involving human participants were reviewed and approved by the Sichuan Regional Ethics Review Committee on Traditional Chinese Medicine (ID: 2015KL-002). The patients/participants provided their written informed consent to participate in this study.

## Author contributions

FZ and TY conceived and designed the study. RS, ZH, SY, PM, YQ, and LC recruited the participants. PZ and YM analyzed the data. PZ drafted the manuscript. RS participated in MRI scan design and image collection. FZ revised the manuscript. All authors contributed to the article and approved the submitted version.

## Footnotes

1
http://www.fil.ion.ucl.ac.uk/spm

2
http://rfmri.org/DPARSF

3
https://www.nitrc.org/projects/gretna

4
https://www.nitrc.org/projects/bnv
