# Supplementary material for: Aberrant resting-state functional connectivity and topological properties of the subcortical network in functional dyspepsia patients
Source: Front Mol Neurosci. 2022 Oct 13;15:1001557. doi: 10.3389/fnmol.2022.1001557 (PMC9606653; doi:10.3389/fnmol.2022.1001557)
Supplement: Supplementary file 1 [file Data_Sheet_1.docx]

**Supplementary material 1**

**The coordinate of the subcortical regions in nomenclature for 3T parcellation hierarchy.**

| **Regions** | **Coordinate** | | |
| --- | --- | --- | --- |
|  | **X** | **Y** | **Z** |
| HIP_R | 28.0 | -22.0 | -14.0 |
| AMY_R | 24.0 | -4.0 | -18.0 |
| pTHA_R | 16.0 | -26.0 | 2.0 |
| aTHA_R | 10.0 | -14.0 | 8.0 |
| NAc_R | 12.0 | 14.0 | -6.0 |
| GP_R | 20.0 | -4.0 | -2.0 |
| PUT_R | 26.0 | 0.0 | 0.0 |
| CAU_R | 14.0 | 10.0 | 10.0 |
| HIP_L | -26.0 | -22.0 | -14.0 |
| AMY_L | -22.0 | -4.0 | -18.0 |
| pTHA_L | -14.0 | -26.0 | 2.0 |
| aTHA_L | -8.0 | -14.0 | 8.0 |
| NAc_L | -10.0 | 14.0 | -6.0 |
| GP_L | -18.0 | -4.0 | -2.0 |
| PUT_L | -24.0 | 0.0 | 0.0 |
| CAU_L | -12.0 | 10.0 | 10.0 |

**Abbreviations：** HIP_R, right head of hippocampus; AMY_R, right amygdala; pTHA_R, right posterior thalamus; aTHA_R, right anterior thalamus; NAc_R, right nucleus accumbens; GP_R, right globus pallidus; PUT_R, right putamen; CAU_R, right caudate nucleus; HIP_L, left hippocampus; AMY_L, left amygdala; pTHA_L, left posterior thalamus; aTHA_L, left anterior thalamus; NAc_L, left nucleus accumbens; GP_L, left globus pallidus; PUT_L, left putamen; CAU_L, left caudate nucleus.

**Supplementary material 1**


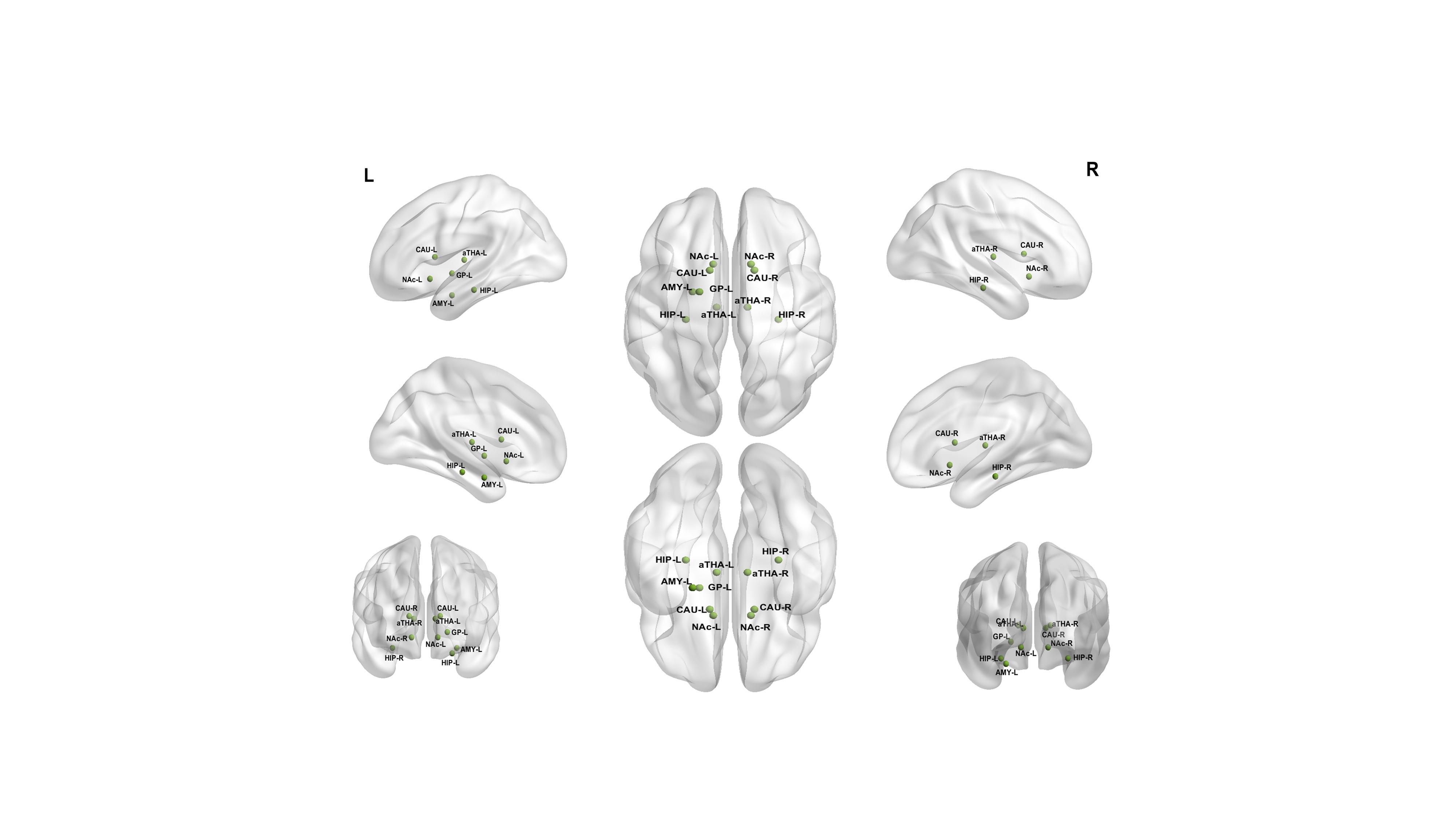


**Supplementary material 2**

**Global Level Properties**

***The Clustering coefficient***

The clustering coefficient (*Cp*) quantifies the number of connections that exist between the nearest neighbors of a node as a proportion of the maximum number of possible connections. For a given node *i*, $C_{i}$ is defined as [1]:

$$C_{i}=\frac{2e_{i}}{k_{i}(k_{i}-1)}$$

where $k_{i}$ is the degree of node *i*, and $e_{i}$ is the number of actual existing connections between the nearest neighbors of node *i*. $C_{i}$ is a ratio of the total number of edges divided by the maximum possible number of edges between the nearest neighbors of node *i*. The mean clustering coefficient of a network $C_{p}$ is the average of the clustering coefficient over all nodes in a network:

$$C_{P}=\frac{1}{N}C_{i}$$

***The shortest path length***

The shortest path length (*Lp*) reflects the mean minimal travel path between any pair of nodes (e.g., node *i* and node *j*) in the network [2]. The shortest path length of a network is defined as:

$$L_{p}=\frac{1}{N(N-1)}\sum_{i\neq j\in G} L_{ij}$$

where *N* is the number of nodes in the network, $L_{ij}$ is defined as the shortest path length between node i and node j in the network. The shortest path length of a network quantifies the average number of connections between nodes along the shortest paths.

***The normalized clustering coefficient, normalized shortest path length, and small-worldness***

To compute the small-world properties of the network, the values of $C_{p}$ and $L_{p}$ were compared with 100 matched random networks. The random networks preserve the same number of nodes, edges, and degree distribution as real networks [3]. Furthermore, the normalized clustering coefficient (*γ*) and normalized shortest path length (*λ*) were calculated as follows:

$$\gamma=\frac{C_{p}^{real}}{C_{p}^{rand}}$$

$$\lambda=\frac{L_{p}^{real}}{L_{p}^{rand}}$$

where $C_{p}^{rand}$ and $L_{p}^{rand}$ are the mean clustering coefficient and shortest path length of 100 matched random networks. A network is considered to have small-world properties if *γ* > 1 and *λ* ≈1. Then, these two parameters were unified into a simple quantitative parameter, small-worldness (*σ*). The small-worldness of a network is defined as:

$$\sigma=\frac{\gamma}{\lambda}$$

A real network is said to be small-world if *σ* > 1, and has a stronger small-world property when the value of *σ* is higher.

***The global efficiency***

*The global efficiency* ($E_{glob}$) of network G measures the ability of parallel information transmission in the network [2], which is computed as follows:

$$E_{glob}(G)=\frac{1}{N(N-1)}\sum_{i\neq j\in G} \frac{1}{L_{ij}}$$

where $L_{ij}$ is the shortest path length between node *i* and node *j* in network G.

***The local efficiency***

The local efficiency ($E_{loc}$) of network G measures how much the network is fault-tolerant and shows the capability of information transfer in each subgraph when node *i* is removed [2], which is defined as:

$$E_{loc}(G)=\frac{1}{N}\sum_{i\in G} E_{glob}(G_{i})$$

where $E_{glob}$ is the global efficiency of the network, and $G_{i}$ denotes the subgraph composed of the nearest neighbors of node *i*.

**Nodal Level Properties**

***The nodal degree centrality***

The nodal degree is defined as the number (binary graph) or the total connectivity strength (weight graph) of all connections that link to a node, reflecting the centrality of this node in the network [4].

$$D_{i}=\sum_{j=1}^{n} a_{ij}$$

***The nodal efficiency***

The nodal efficiency measures the mean shortest path length between a given node *i* and all of the other nodes in the network [5], which is defined as follows:

$$E_{nodal}=\frac{1}{N-1}\sum_{i\neq j\in G} \frac{1}{L_{ij}}$$

where $L_{ij}$ is the shortest path length between node *i* and node *j* in network G.

**Supplementary material 3**

**The strengthed rsFCs of subcortical networks among FD patients**

**
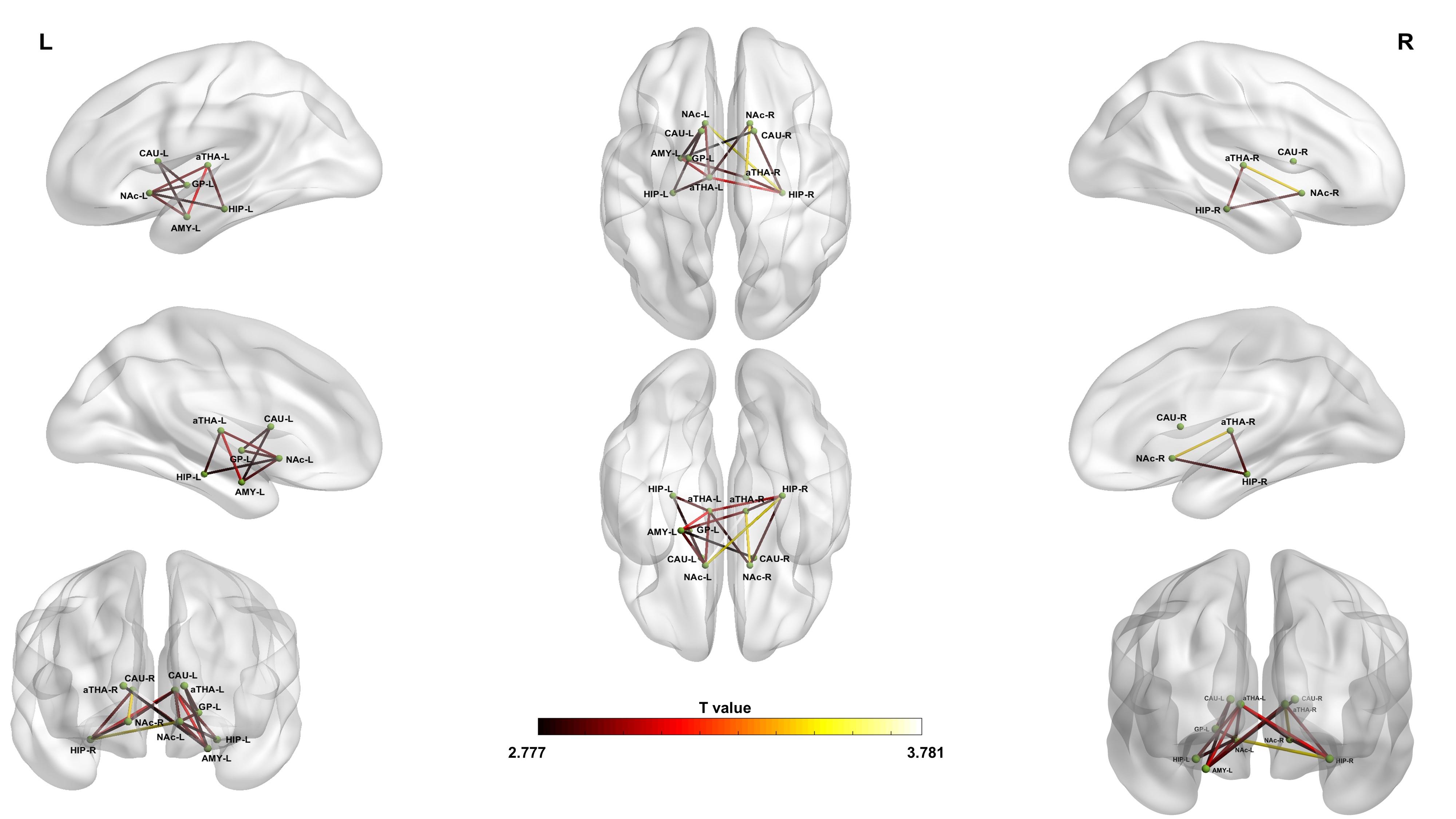
**

**Abbreviations:** rsFC, rest state functional connectivity**;** HIP_R, right hippocampus; AMY_R, right amygdala; pTHA_R, right posterior thalamus; aTHA_R, right anterior thalamus; NAc-R, right nucleus accumbens; GP-R, right globus pallidus; PUT-R, right putamen; CAU_R, right caudate nucleus; HIP_L, left hippocampus; AMY_L, left amygdala; pTHA_L, left posterior thalamus; aTHA_L, left anterior thalamus; NAc_L, left nucleus accumbens; GP_L, left globus pallidus; PUT_L, left putamen; CAU_L, left caudate nucleus. ^*^The marker indicated a significant difference between the FD group and the HC group.

**Supplementary material 4**

**The key small-world parameters of the subcortical networks in the defined sparsity**


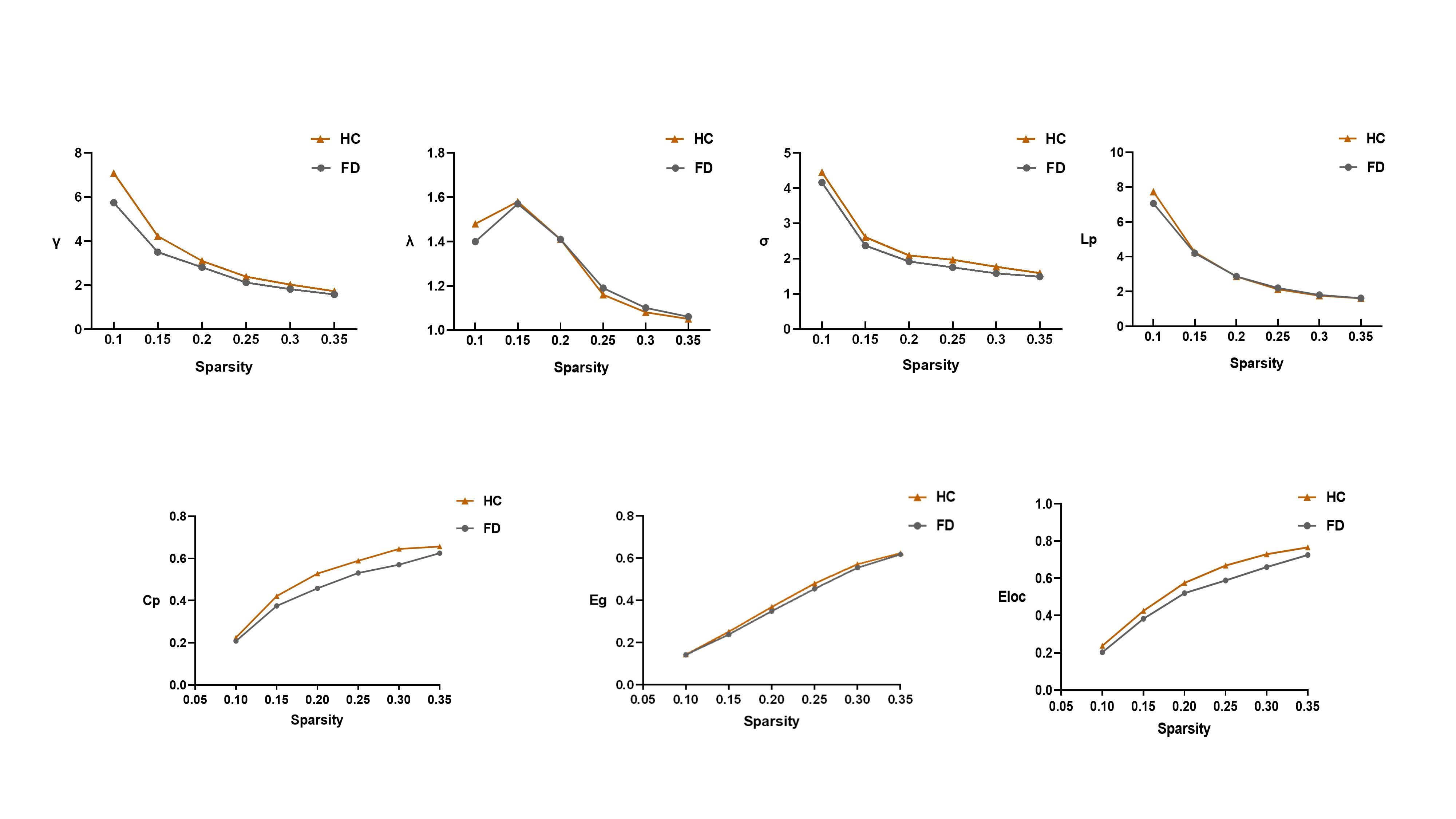


**Supplementary Figures 1:** The key small-world parameters of the subcortical networks in the defined sparsity threshold. figures showed that in the defined threshold range, both FD and HC groups exhibited small-world network architecture in the subcortical networks (γ > 1, λ ≈ 1 and σ = γ/λ > 1), which indicated that both groups exhibited the features of small-world topology. γ, normalized clustering coefficient; λ, normalized characteristic path length; σ, small-worldness.

**Supplementary material 5**

1. **Retest of the results of global network metrics in FD patients via Non-Paramateric test (Mann-Whitney U).**


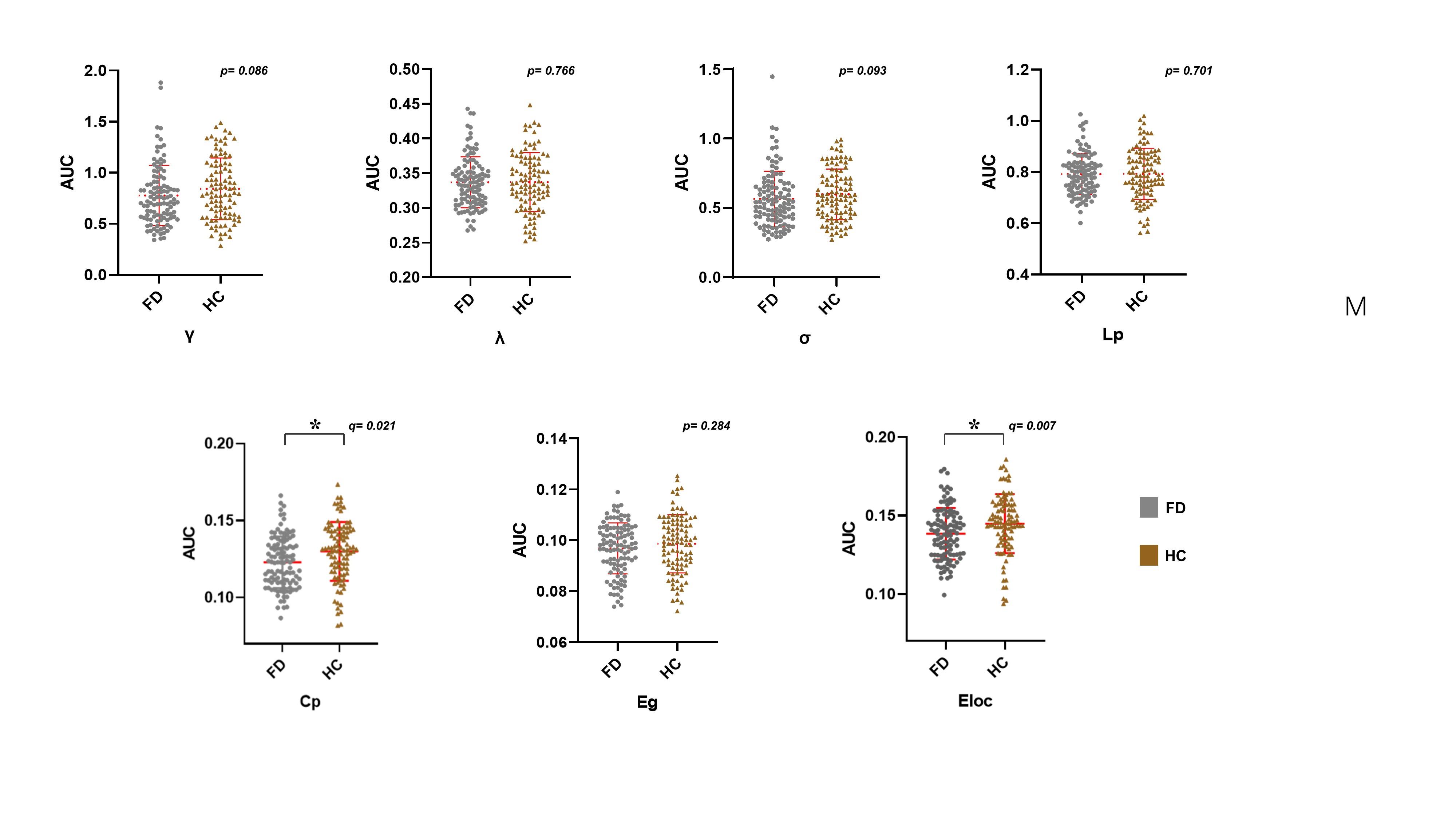


1. **Retest of the results of nodal properties in FD patients via Non-Paramateric test (Mann-Whitney U).**

**
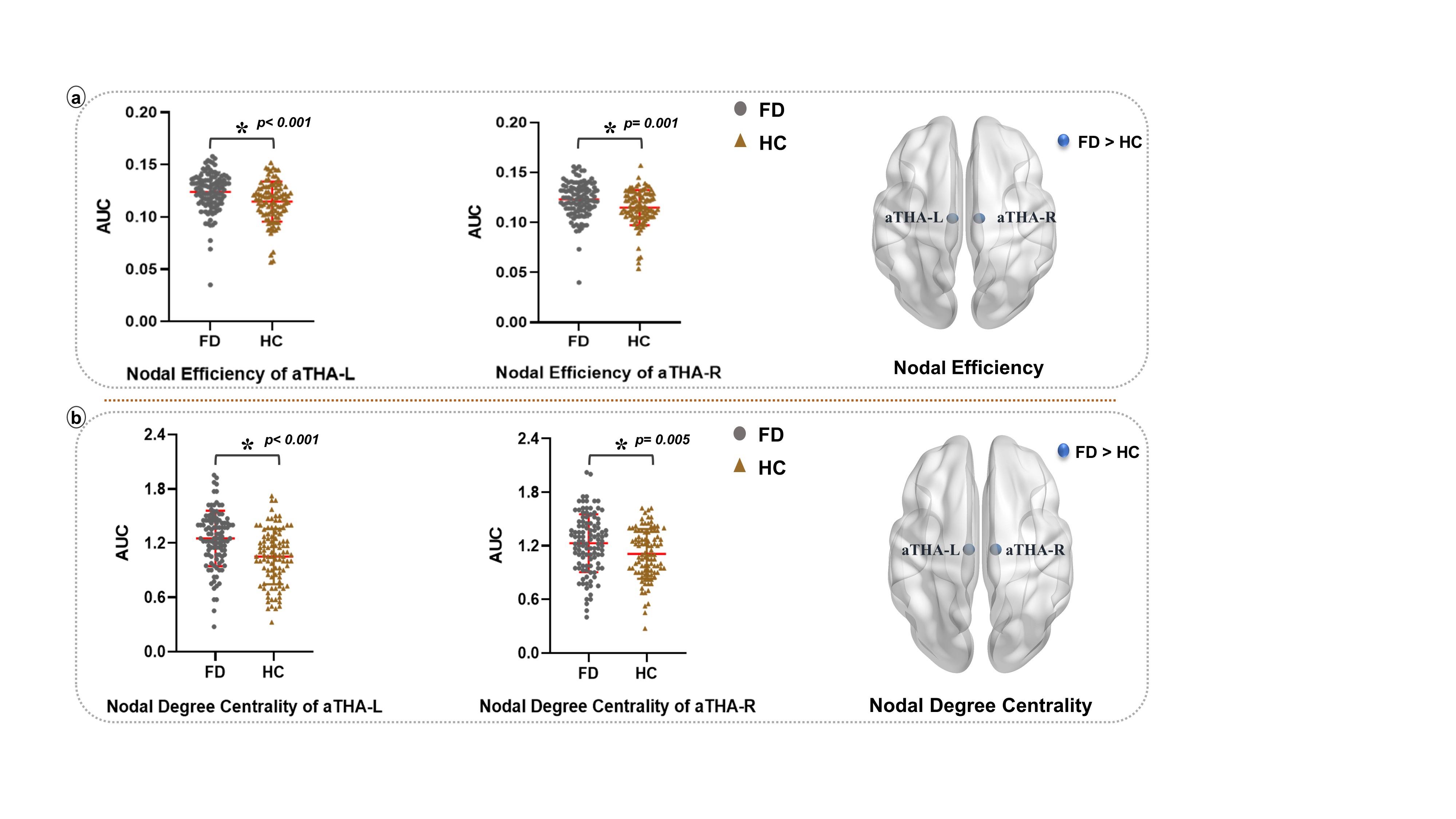
**

1. **Formula false discovery rate (FDR) correction**

We used Benjamini–Hochberg procedure to do FDR correction [6]. The settings for FDR procedures is such that we have *H_1_…H_m_* null hypotheses tested and *P_1_…P_m_* their corresponding [*p-values*](https://en.wikipedia.org/wiki/P-value). We list these *p-values* in ascending order and denote them by P(1)…P(m). A procedure that goes from a small p-value to a large one will be called a step-up procedure. In a similar way, in a "step-down" procedure we move from a large corresponding test statistic to a smaller one.

The Benjamini–Hochberg procedure (BH step-up procedure) controls the FDR at level α. It works as follows:

1. For a given α, find the largest k such that $p_{(k)}\leq\frac{k}{m}\alpha$.
2. Reject the null hypothesis (i.e., declare discoveries) for all *H(i)* for *i*=*1,2….,k*.

**Reference**

1. Watts DJ, Strogatz SH (1998) Collective dynamics of “small-world” networks. Nature 393:440–442. https://doi.org/10.1038/30918

2. Nan J, Zhang L, Zhu F, et al (2016) Topological Alterations of the Intrinsic Brain Network in Patients with Functional Dyspepsia. J Neurogastroenterol Motil 22:118–128. https://doi.org/10.5056/jnm15118

3. Maslov S, Sneppen K (2002) Specificity and stability in topology of protein networks. Science 296:910–913. https://doi.org/10.1126/science.1065103

4. Zuo X-N, Ehmke R, Mennes M, et al (2012) Network centrality in the human functional connectome. Cereb Cortex 22:1862–1875. https://doi.org/10.1093/cercor/bhr269

5. Achard S, Bullmore E (2007) Efficiency and cost of economical brain functional networks. PLoS Comput Biol 3:e17. https://doi.org/10.1371/journal.pcbi.0030017

6. Benjamini Y, Hochberg Y (1995) Controlling the false discovery rate: a practical and powerful approach to multiple testing. Journal of the Royal statistical society: series B (Methodological) 57:289–300
